# Polymorphisms and environmental factors associated with orofacial clefts as potential markers for oral cancer risk

**DOI:** 10.1590/1807-3107bor-2025.vol39.089

**Published:** 2025-09-15

**Authors:** Adriana Mendonça da SILVA, Michelle Miranda Lopes FALCÃO, Valéria Souza FREITAS, Alexandre Resende VIEIRA

**Affiliations:** (a) Universidade Estadual de Feira de Santana –UEFS, Department of Health, Feira de Santana, BA, Brazil.; (b) East Carolina University, School of Dental Medicine, Greenville, NC, USA.

**Keywords:** Leukoplakia, Oral, Lichen Planus, Squamous Cell Carcinoma of Head and Neck, Environmental Exposure, Polymorphism, Single Nucleotide

## Abstract

The etiological intersection between orofacial clefts and oral cancer may involve environmental factors modulating gene expression in shared biological pathways. This study aimed to investigate the association between orofacial clefts and oral potentially malignant disorders or oral squamous cell carcinoma, focusing on genetic variants and environmental risk factors. A case-control design was employed, comprising 48 histologically confirmed cases of oral potentially malignant disorders or oral squamous cell carcinoma and 96 age- and sex-matched controls. Information on family history of orofacial cleft, and biological and environmental risk factors, was collected through interviews. Genomic DNA was extracted from saliva samples and genotyped for rs1533767 *(WNT11)*, rs9879992 (*GSK3B*), and rs3923087 and rs11867417 (*AXIN2*). Unadjusted and adjusted odds ratios (OR) for the associations between family history of orofacial cleft and oral potentially malignant disorders/oral cancer, and between environmental risk factors and oral potentially malignant disorders/oral cancer were calculated using STATA software. Genotype and allele frequency comparisons between groups were conducted using PLINK Software. Statistical significance was defined as p<0.05 and 95% confidence interval (95%CI). No statistically significant association was found between family history and orofacial clefts (p = 0.52). However, place of residence (adjusted OR:5.46, p < 0.001, 95%CI: 3.76–63.543), and three genetic variants—rs1533767 (OR: 1.94, p = 0.042, 95%CI: 1.018–3.694), rs3923087 (OR: 0.58, p = 0.038, 95%CI: 0.344–0.974), rs11867417 (OR: 0.51, p = 0.010, 95%CI: 0.304–0.857)—were associated with oral potentially malignant disorders and oral squamous cell carcinoma. These findings suggest that specific environmental risk factors and genetic variants may be associated with increased susceptibility to oral potentially malignant disorders and oral cancer.

## Introduction

Orofacial clefts (OC) are the most common craniofacial anomalies worldwide.^1^ They affect over 10 million people, and it is estimated that a new case occurs every three minutes across the globe.^2^ Its global prevalence is 1 in 700 live births, with variations according to geography, socioeconomic status, race, and sex.^3^ OC represent a significant public health concern, given their aesthetic, functional, and emotional impacts, as well as their contribution to increased morbidity and mortality.^3^

Oral cancer similarly constitutes a global public health issue.^4^ In 2020, approximately 370,000 new cases and 170,000 deaths due to lip and oral cavity cancers were reported worldwide.^5^ In Brazil alone, an estimated 10,900 new cases in men and 4,200 in women were expected in 2023.^6^ In about 90% of cases, oral cancer presents as oral squamous cell carcinoma (OSCC),^7^ which is typically preceded by oral potentially malignant disorders (OPMD).

OPMD encompass a group of epithelial lesions that exhibit histopathological features of oral epithelial dysplasia and carry a higher risk of malignant transformation into OSCC compared to normal epithelium^8^. Oral leukoplakia is the most commonly reported OPMD in the oral cavity, with a general prevalence of approximately 2%, which increases with age. Other phenotypes include erythroplakia and oral lichen planus, with mean prevalence rates of 0.11% and 1%, respectively.^8^

These disorders have a multifactorial etiology, characterized by a complex interaction between genetic and environmental factors, which complicates the understanding of their underlying mechanisms.^9^ The etiological intersection between OC and oral cancer may be explained by environmental factors capable of modulating gene expression in shared biological pathways.^10^ Such factors may predispose individuals to OC when alterations occur during embryonic development and to OPMD and/or oral cancer when they occur later in life.

The hypothesis that OC and cancer may share a common etiology^11^has been supported by studies identifying single nucleotide polymorphisms (SNP) simultaneously associated with both conditions,^12^ particularly within the Wnt gene family—a genetic pathway essential for cellular growth and differentiation.^13^ Genetic alterations in this pathway have been proposed to explain the association between OC and OSCC,^14,15^ considering that disruptions in cell migration, proliferation, differentiation, and apoptosis are closely linked to the development of both conditions.^16^ Furthermore, several oral lesions classified as OPMD demonstrate alterations in molecular mechanisms regulated by Wnt pathway genes.^17^ In addition, environmental factors—such as smoking, alcohol consumption, limited access to high-quality primary care, racism, and socioeconomic disparities—may exacerbate genetic risk variants,^18^ influence health status through biological mechanisms,^19^ and increase susceptibility to both OC and cancer.

Despite the biological plausibility, few studies have investigated the association between OC and oral cancer^14,15^ or between OC and OPMD.^20^ Genetic variants are inherited from one’s parents and are not easily modified. However, many environmental exposures are modifiable or preventable through public policy initiatives and changes in individual behavior. A deeper understanding of how genetic and environmental factors interact is essential for effectively addressing these diseases.^18^ In this context, the objective of the present study was to investigate the association between OC and OPMD/OSCC, with a focus on genetic variations and environmental risk factors.

## Methods

This case-control study included 144 participants receiving treatment at the dental clinics of the State University of Feira de Santana, Brazil. All participants provided written informed consent. The study was approved by the ethics committee of the State University of Feira de Santana (approval number: 61386322.8.0000.0053) and the University of Pittsburgh (IRB number: 03829018.1.0000.5183). The study followed the STROBE and STREGA reporting guidelines.

The case group consisted of all adult patients (n = 48) of both sexes with a confirmed histopathologic diagnosis of OPMD (12 with oral leukoplakia and 15 with oral lichen planus) or OSCC (21 with oral cancer) who were treated at the Oral Pathology Clinic of the university during the recruitment period. Individuals who had previously been treated for these conditions or were considered to be in remission for at least six months, as well as those currently undergoing treatment, were also included. Exclusion criteria were a history of cancer in anatomical sites other than the oral cavity, speech impairments, or difficulties in saliva collection due to hyposalivation.

The control group consisted of unrelated adult subjects (n = 96) of both sexes, with no history of OPMD or cancer. Controls were matched to cases by sex and age in a 1:2 ratio and were selected from the same reference population as the cases to ensure greater comparability (Figure). Individuals with a history of cancer or OPMD in any anatomical site, as well as relatives or household members of eligible case participants, were excluded from the control group. This exclusion aimed to minimize potential bias arising from shared lifestyle habits influenced by social interactions.

**Figure:**
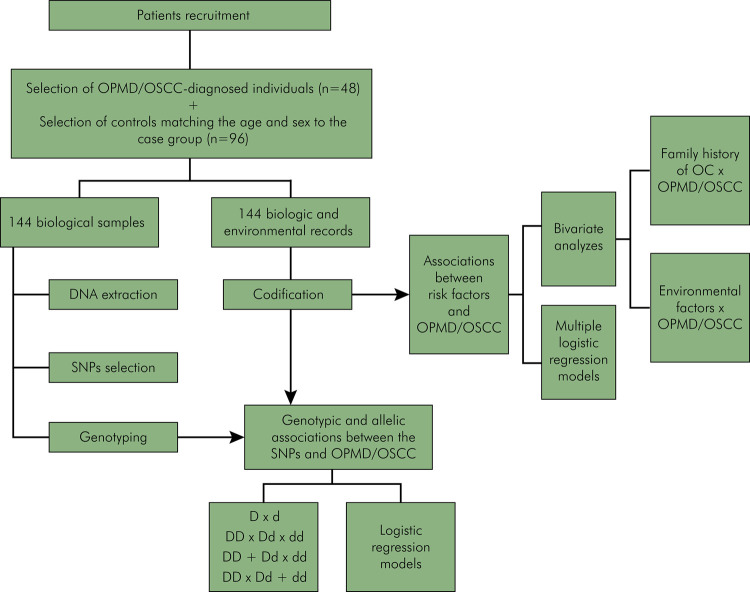
Overall study design.

Information on biological and environmental factors was obtained through interviews conducted by a single researcher, using a structured questionnaire addressing the following domains: a) sociodemographic factors (sex, age, ethnicity, education, marital status, occupation and place of residence); b) behavioral and lifestyle factors (smoking and alcohol consumption); and c) family history of OC and cancer. Participants who were unable to report information on family history of OC or cancer were excluded.

Following the interview, participants underwent clinical evaluation by a single dentist specializing in oral pathology to assess the presence of OPMD or OSCC, based on the diagnostic criteria established by the World Health Organization in its Classification of Head and Neck Tumours.^21^ Individuals with a clinical diagnosis of these conditions subsequently underwent biopsy to confirm the diagnosis. Regarding participants who had a prior history of or who were undergoing treatment for these conditions, any information on the histological type of the oral lesion, its anatomical location, staging, and previous treatments was retrieved from clinical records.

The same dentist also conducted a clinical evaluation to assess tooth loss due to either agenesis or extraction. Agenesis was diagnosed by visual inspection when one or more teeth were absent in the oral cavity, according to the normal chronology of dental development, and in the absence of a reported history of extraction or trauma-related tooth loss.^22^ Individuals with a history of tooth extraction were excluded from the agenesis analysis, since it was not possible to rule out congenital absence in those cases.^22^

Finally, saliva collection for DNA isolation was performed at the same location as the dental examination by the same dentist. Participants were asked to provide approximately 4 mL of unstimulated saliva into a sterile 10 mL collection tube over a 15-minute period. The saliva was then transferred into Eppendorf tubes (2 ml) labeled according to the sample number and immediately stored at -20^o^ until genotyping analysis. All genetic analyses were conducted blind to the participants’ clinical diagnoses.

For genotyping, four SNPs located in three Wnt pathway genes were selected based on prior associations with OC and oral cancer:^14,15^ rs1533767 *(WNT11)*, rs9879992 (*GSK3B*), rs3923087 and rs11867417 (both in *AXIN2*). Genotyping began with sample preparation, in which DNA was extracted using the ammonium acetate protocol^23^. Subsequently, real-time polymerase chain reactions (PCR) were carried out using the DNA samples and a reaction mixture containing the SNP-specific primers, TaqMan probes, and a master mix^24^ in the GeneAmp PCR System 9700 (Applied Biosystems, Foster City, USA). Allelic discrimination for each sample was determined using the QuantStudio 6 Flex system (Applied Biosystems, Foster City, USA). PCR reactions were repeated when necessary, and the acceptable range for missing data varied from 6.9% (rs11867417) to 12.5% (rs1533767).

The dependent variable was the presence of OPMD or OSCC. The independent variables included family history of OC (for the epidemiological analysis) and the selected SNPs (for the genetic analysis). Covariates considered in the analyses were sex, age, ethnicity, educational level, consanguinity, occupation, place of residence, family history of cancer, agenesis, and alcohol and tobacco use (Table 1).

**Table 1:** Description of the study variables.

Variable	Description
Biological characteristics	
Family history of orofacial clefts	Yes, No
Single nucleotide polymorphisms	rs1533767 (*WNT11*), rs9879992 (*GSK3B*), rs3923087 and rs11867417 (both in *AXIN2*)
Sex	Female, Male
Age	Continuous
Ethnicity	White, Brown, Black
Consanguinity	Yes, No
Family history of cancer	Yes, No
Behavioral/ lifestyle characteristics	
History of dental agenesis/extraction	Yes, No
Number of missing teeth	Continuous
Smoking habits	Yes, No
Type of smoking	Cigarette, Cigarette and Other, Other
Alcohol consumption habits	Yes, No
Type of alcoholic beverage	Fermented, Distilled, Both
Socioeconomic characteristics	
Marital status	Single, Married, Divorced, Widowed
Place of residence	Urban, Rural
Education	Illiterate, Elementary school, High school, Graduate
Occupation	Agricultural worker, Other

For epidemiologic analyses, chi-square and Fisher’s exact tests were used to compare categorical variables between cases and controls. Bivariate associations between family history of OC and OPMD/OSCC, as well as between environmental risk factors and OPMD/OSCC, were assessed using odds ratios (OR). Adjusted ORs for these associations were estimated using multiple logistic regression models. All epidemiological analyses were performed using STATA software, with statistical significance defined as p < 0.05 and a 95% confidence interval (95%CI).

For genetic analyses, Hardy–Weinberg equilibrium was assessed using the chi-square test, with a statistical threshold of p > 0.012 (calculated as 0.05 divided by the number of markers, n = 4). Genotype and allele frequencies in cases and controls were determined, considering d as the major allele and D the minor allele. The genetic models tested included: allelic (D vs. d), genotypic (DD vs. Dd vs. dd), dominant (DD + Dd vs. dd), and recessive (DD vs. Dd + dd).^25^ Logistic regression models were used to estimate adjusted ORs for associations between the alleles and clinical outcomes. All genetic analyses were conducted using PLINK software, with statistical significance set at p < 0.05 and a 95%CI.

## Results

### Biological, behavioral/lifestyle, and socioeconomic profile

The mean age of participants was 60 years (± 15) overall, with similar distributions between groups [cases: 60.3 (± 15.6); controls: 60.5 (± 15); p = 0.436], ranging from 22 to 87 years. Most participants were male (54.8%) and self-identified as brown (48.6%). Few individuals reported consanguinity, with one case at the third-degree level and nine at the fourth-degree level. A majority of participants (n = 74; 51.9%) reported no family history of cancer. There were no statistically significant differences between cases and controls regarding biological characteristics.

Both groups reported a high prevalence of dental extractions (93%). However, the mean number of missing teeth differed significantly between cases and controls (p = 0.001), with cases missing an average of 16 teeth (± 12) and controls, 13 (± 9). Edentulism was more prevalent among cases, whereas controls tended to have fewer missing teeth (p = 0.006). A total of 54.9% of participants reported current or past smoking. However, individuals with OPMD/OSCC reported a significantly higher frequency of tobacco use (p = 0.024). Alcohol consumption was also common (65.3%), with fermented beverages being the most frequently consumed type (57.1%).

Regarding socioeconomic characteristics, most participants were married (n = 72), lived in urban areas (n = 115), had completed elementary education (n = 66), and worked in the service sector (n = 59). All socioeconomic covariates differed significantly between groups. Individuals with OPMD/OSCC were more likely to reside in rural areas (p < 0.001), have lower educational attainment (p = 0.04), and work in agriculture (p = 0.006) (Table 2).

**Table 2:** Descriptive statistics and group comparisons for biological, behavioral, and socioeconomic characteristics.

Variables	Total Population	Case	Control	p-value
	n (%)	n (%)	n (%)	
Biological				
Sex				1.000
Female	69 (45.2)	23 (47.9)	46 (47.9)	
Male	75 (54.8)	25 (52.1)	50 (52.1)	
Ethnicity				0.971
White	25 (17.4)	8 (16.7)	17 (17.7)	
Brown	70 (48.6)	24 (50.0)	46 (47.9)	
Black	49 (34.0)	16 (33.3)	33 (34.3)	
Consanguinity				0.854
No	131 (92.9)	43 (93.5)	88 (92.6)	
Yes	10 (7.1)	3 (6.5)	7 (7.4)	
Family history of cancer				0.099
No	74 (51.4)	20 (41.7)	54 (56.2)	
Yes	70 (48.6)	28 (58.3)	42 (43.7)	
Family history of orofacial clefts				0.520
No	139 (96.5)	47 (97.9)	92 (95.8)	
Yes	5 (3.5)	1 (2.1)	4 (4.2)	
Behavioral/lifestyle				
History of extraction				0.816
No	10 (7.0)	3 (6.3)	7 (9.3)	
Yes	134 (93.0)	45 (93.7)	89 (92.7)	
Number of missing teeth (categorical)				**0.006**
1-5 teeth	36 (25.3)	11 (23.4)	25 (26.4)	
6-15 teeth	47 (33.1)	14 (29.7)	33 (34.7)	
16-31 teeth	44 (31.0)	11 (23.4)	33 (34.7)	
Edentulous	14 (10.6)	11 (23.4)	4 (4.2)	
Smoking habits				**0.024**
No	79 (54.9)	20 (41.7)	59 (61.5)	
Yes	65 (45.1)	28 (58.3)	37 (38.5)	
Type of smoking				**0.032**
Cigarette	51 (77.3)	18 (64.3)	33 (86.8)	
Cigarette and other	5 (7.6)	2 (7.1)	3 (7.9)	
Others	10 (15.1)	8 (28.6)	2 (5.26)	
Alcohol consumption habits				0.536
No	50 (34.7)	15 (31.2)	35 (36.5)	
Yes	94 (65.3)	33 (68.3)	61 (63.5)	
Type of alcoholic beverage				0.536
Fermented	52 (57.1)	14 (43.7)	38 (64.4)	
Distilled	19 (20.9)	11 (34.4)	8 (13.6)	
Both	20 (22.0)	7 (21.9)	13 (22.1)	
Socioeconomic				
Place of residence				**0.000**
Urban	115 (80.4)	25 (52.1)	90 (94.7)	
Rural	28 (19.6)	23 (47.9)	5 (5.3)	
Education				**0.040**
Illiterate	17 (12.0)	11 (22.9)	6 (6.4)	
Elementary school	66 (46.5)	19 (39.6)	47 (50.0)	
High school	42 (29.5)	13 (27.1)	29 (30.8)	
Graduate	17 (12.0)	5 (10.4)	12 (12.7)	
Occupation				**0.006**
Agricultural worker	26 (18.1)	17 (35.4)	9 (9.4)	
Service worker	59 (40.9)	17 (35.4)	42 (43.8)	
Mid-level technician	7 (4.9)	2 (4.2)	5 (5.2)	
Industrial production worker	3 (2.1)	0 (0.0)	3 (3.1)	
Construction worker	12 (8.3)	1 (2.1)	11 (11.5)	
Science professional	13 (9.0)	3 (6.2)	10)10.4)	
Administrative service worker	3 (2.1)	2 (4.2)	1 (1.0)	
Other	21 (14.6)	6 (12.5)	15 (15.6)	

p-values calculated using chi-square tests. Bold indicates statistically significant p < 0.05

### Association between OC and OPMD/OSCC

None of the participants in either group had a personal history of OC. Five individuals reported a family history of OC (cases: n = 1; controls: n = 4), with relationships classified as second-degree (cases: n =1; controls: n = 1) and third-degree (controls: n = 3). The chi-square test showed no statistically significant difference between the groups regarding family history of OC (p = 0.52). Likewise, both bivariate analysis and logistic regression failed to identify significant associations (Table 3).

**Table 3:** Unadjusted and adjusted odds ratios for environmental factors associated with OPMD/OSCC.

Exposure	OR	p-value (95%CI)	Adjusted OR	p-value (95%CI)
Family history of orofacial cleft	0.49	0.528 (0.531–4.0502)	0.88	0.913 (0877–8.804)
Place of residence (rural)	16.56	**0.000** (5.315–59.992)	15.46	**0.000** (3.76–63.543)
Smoking habits (smokers)	2.13	**0.033** (1.056–4.323)	1.92	0.118 (0.847–4.358)
Occupation (agriculture)	5.30	**0.001** (1.970–14.813)	0.77	0.740 (0.176–3.427)
Education (illiterate)	4.36	**0.004** (1.344–15.309)	1.78	0.419 (0.438–7.264)

OR: odds ratio; CI: confidence interval. Bold indicates statistically significant p < 0.05; “Place of residence (rural)” indicates rural vs. urban.

### Association between environmental factors and OPMD/OSCC

Bivariate analysis revealed statistically significant differences in the distribution of environmental variables between groups. However, after adjustment in the multiple logistic regression model, only place of residence remained significantly associated with OPMD/OSCC (adjusted OR = 15.46, p < 0.001, 95% CI: 3.76–63.54) (Table 3). Model performance was assessed through the goodness-of-fit and explanatory power measures. The Hosmer–Lemeshow test (p = 0.53) indicated an adequate fit, suggesting that predicted values aligned well with the observed outcomes. The area under the receiver operating characteristic (ROC) curve (AUC = 0.756) suggested that the model demonstrated good predictive accuracy for the dependent variable based on the independent variables. In addition, the linearity of non-dichotomous predictors (p = 0.676) confirmed a linear relationship between these variables and the logit of the outcome.

### Identification of SNPs and their association with OPMD/OSCC

The distribution of genotypes was consistent with Hardy–Weinberg equilibrium among the cases for all four SNPs: *GSK3B* rs9879992 (p = 0.51), WNT11 rs1533767 (p = 0.70), *AXIN2* rs3923087 (p = 0.211), and *AXIN2* rs11867417 (p = 0.525). This indicates that there were no statistically significant differences between the observed and the expected genotype frequencies under Hardy–Weinberg equilibrium. In contrast, a deviation from this equilibrium was observed for *AXIN2* rs3923087 in the control group (p < 0.001) (Table 4).

**Table 4:** Hardy–Weinberg equilibrium for each SNP by group.

Gene (SNP)	Allele D/d	Group	Observed heterozygosity	Expected heterozygosity	HWE
					(p-value)
*GSK3B* (rs9879992)	G/A	Affected	0.40	0.44	0.510
		Unaffected	0.42	0.48	0.267
*WNT11* (rs1533767)	A/G	Affected	0.41	0.38	0.700
		Unaffected	0.20	0.25	0.078
*AXIN2* (rs3923087)	C/T	Affected	0.37	0.46	0.211
		Unaffected	0.30	0.50	0.000
*AXIN2* (rs11867417)	C/T	Affected	0.41	0.46	0.525
		Unaffected	0.37	0.50	0.019

HWE: Hardy–Weinberg equilibrium; D: minor allele; d: major allele. Bold indicates deviation from HWE at p < 0.012, Bonferroni correction for 4 comparisons.

Several associations were identified between the phenotypes and the selected SNPs (Table 5). WNT11 rs1533767 was significantly associated with OPMD/OSCC in the genotypic model (p = 0.038), as well as in the allelic (OR: 1.94, p = 0.042, 95% CI: 1.018–3.694) and dominant models (OR: 1.94, p = 0.042, 95%CI: 1.018–3.694). The two *AXIN2* SNPs—rsrs3923087 and rs11867417—also showed significant associations with OPMD/OSCC in both the allelic (OR: 0.58, p = 0.03845, 95%CI: 0.344–0.974; and OR: 0.51, p = 0.010, 95%CI: 0.304–0.857, respectively) and recessive models (OR: 0.40, p = 0.04, 95%CI: 0.168–0.975; and OR: 0.35, p = 0.022, 95%CI: 0.141–0.883, respectively). In contrast, *GSK3B* rs9879992 was not significantly associated with OPMD/OSCC under any of the tested models. Logistic regression models adjusted for covariates were performed for WNT11 rs1533767 and *AXIN2* rs3923087 and rs11867417 (Table 6).

**Table 5:** Association tests between SNPs and OPMD/OSCC across genetic models.

Gene (SNPs)	Allele D/d	MAF	Model	Affected	Unaffected	Chi-square value	Degree of freedom	p-value	OR (95%CI)
*GSK3B* (rs9879992)	G/A	0.38	Genotypic	6/18/21	16/36/33	0.999	2	0.607	
			Allele	30/60	68/102	1.114	1	0.291	0.75 (0.273–0.4393)
			Dominant	24/21	52/33	0.745	1	0.427	0.72 (0.349–1.505)
			Recessive	6/39	16/69	0.631	1	0.427	0.66 (0.439–1.281)
*WNT11* (rs1533767)	A/G	0.19	Genotypic	2/18/23	4/17/62	6.551	1	**0.038**	
			Allele	22/64	25/141	4.133	1	**0.042**	**1.94 (1.018–3.694)**
			Dominant	20/23	21/62	5.805	1	**0.016**	**2.57 (1.180–5.585)**
			Recessive	2/41	abr/79	0.002	1	1.000	0.96 (0.168–0.975)
*AXIN2 (rs3923087)*	C/T	0.45	Genotypic	8/17/20	31/27/31	4.216	2	0.1215	
			Allele	33/57	89/89	4.285	1	**0.038**	**0.58 (0.344–0.974)**
			Dominant	25/20	58/21	1.172	1	0.279	0.69 (0.321–1.389)
			Recessive	8/37	31/58	4.213	1	**0.040**	**0.40 (0.168–0.975)**
*AXIN2 (rs11867417)*	C/T	0.47	Genotypic	7/19/20	30/33/26	5.735	2	0.056	
			Allele	33/59	93/85	6.536	1	**0.010**	**0.51 (0.304–0.857)**
			Dominant	26/20	63/26	2.747	1	0.097	0.53 (0.256–1.125)
			Recessive	7/39	30/59	5.211	1	**0.022**	**0.35 (0.141–0.883)**

MAF: minor allele frequency; OR: odds ratio; CI: confidence interval. Genetic models: allelic (D vs. d), genotypic (DD vs. Dd vs. dd), dominant (DD + Dd vs. dd), and recessive (DD vs. Dd + dd), assuming d as the major allele and D as the minor allele. Bold indicates statistically significant p < 0.05.

**Table 6:** Logistic regression models for SNPs adjusted by covariates.

SNP/Minor allele	Adjustment	OR	p-value
rs1533767/A	Smoking habits	1.75	0.091
	Alcohol consumption	1.84	0.059
	Family history of cancer	2.06	**0.029**
	Family history of orofacial cleft	1.82	0.659
	Residence in a rural area	1.90	0.067
rs3923087/C	Smoking habits	0.60	**0.036**
	Alcohol consumption	0.65	0.072
	Family history of cancer	0.69	0.109
	Family history of orofacial cleft	0.64	0.059
	Residence in a rural area	0.66	0.121
rs11867417/C	Smoking habits	0.58	**0.031**
	Alcohol consumption	0.58	**0.026**
	Family history of cancer	0.57	**0.024**
	Family history of orofacial cleft	0.56	**0.019**
	Residence in a rural area	0.59	**0.050**

OR: odds ratio. Bold indicates statistically significant p < 0.05.

## Discussion

The epidemiology analysis revealed no statistically significant difference in the frequency of family history of OC between the case and control groups, suggesting no association between OC and OPMD/OSCC in this study population. Previous studies investigating this relationship did not include formal epidemiological comparisons.^14,15^ In those studies, although a family history of OC was observed among individuals with cancer, the frequency did not differ significantly between groups.^26,27^ Potential explanations for these findings include memory bias,^27^ since families may underreport family history of OC, and limited statistical power due to the small sample sizes.^26^ One systematic review reported an association between cancer and relatives of individuals with OC, but emphasized the need for further research to confirm these findings.^11^

The most commonly studied environmental risk factors for both OC and oral cancer are tobacco use and alcohol consumption.^28,29^ In the present study, no statistically significant association was observed between alcohol consumption and OPMD/OSCC. Although tobacco use was associated with an approximately twofold increased risk of developing OPMD/OSCC, this association did not remain statistically significant after adjustment for potential confounders.

Tooth agenesis has previously been associated with both OC and cancer.^30^ However, we were unable to assess this relationship in the current sample, since many participants already had missing teeth at the time of the examination. Interestingly, a higher frequency of tooth loss and edentulism due to extraction was observed among cases. This finding suggests a possible association between tooth loss and OPMD/OSCC, which is further explored in a separate publication using this dataset.^31^ Since tooth loss or edentulism is not a common risk factor for both OC and cancer, it was not included as a covariate in the logistic regression models.

Regarding socioeconomic factors, living in rural areas was associated with a 15-fold increase in the likelihood of developing OPMD/OSCC. Previous studies have also reported associations between rural residence and increased risk of OC^32^ or other cancers.^33^ A plausible explanation is that individuals living in rural areas may have reduced access to healthcare services or health insurance coverage, which may delay diagnosis and treatment.^32^

The genetic component of this study focused on potential genetic markers previously associated with OC to evaluate their contribution to OPMD/OSCC susceptibility. Three genetic markers—rs1533767, rs3923087, and rs11867417—were found to be significantly associated with OPMD/OSCC. These findings suggest that polymorphisms in WNT11 and *AXIN2*—genes involved in the Wnt signaling pathway and previously related to OC—may also play a role in the development of oral dysplasia and OSCC. This supports earlier evidence suggesting that OPMD carry an inherent risk of malignant transformation.^34^

The SNP WNT11 rs1533767 was statistically associated with OPMD/OSCC. The heterozygous genotype (A/G) was more frequently observed among affected individuals, and the presence of at least one copy of the minor allele A was more common in the case group. This finding suggests an approximately twofold increased risk of disease among carriers of the A allele. Similar results have been reported previously.^14^ In the dominant model, both A/A and A/G genotypes were associated with increased susceptibility to OPMD/OSCC.

Variants in *AXIN2* have previously been associated with cancer, oral leukoplakia, and OC.^35^ In the present study, *AXIN2* rs3923087 and rs11867417 were significantly associated with OPMD/OSCC. The minor allele C was less frequent in cases than in controls for both *AXIN2* variants (rs3923087 and rs11867417), suggesting a potential protective effect. Carriers of at least one copy of the C allele had a reduced risk of developing OPMD/OSCC. Additionally, the recessive models indicated that individuals with the homozygous CC genotype are at lower risk for disease. In contrast, a previous study reported that the T allele of the rs3923087 had a protective effect for OSCC, while no significant association was found for the T allele of rs11867417.^14^ This discrepancy may be attributed to differences in population substructure between samples—such as varying distributions of Northern European and African American ancestry—or to linkage disequilibrium, in which the causal variant lies near but is not same as the SNP under investigation. Moreover, increased expression of *AXIN2* has been strongly correlated with the malignant transformation of oral leukoplakia.^36^

The SNP *AXIN2* rs3923087 exhibited a notable deviation from Hardy–Weinberg equilibrium in the control group (p < 0.001), a finding previously reported in the literature.^37^ Possible explanations include: genetic drift, population structure, or sampling bias specific to this variant.^38^ Additionally, genotyping errors—such as allele miscalling or allelic dropout—may lead to inflated homozygosity (observed distribution: 31/27/31). However, the Hardy–Weinberg test has very low power to detect genotyping errors, particularly when error rates are low and the minor allele frequency is not rare. Small sample sizes may also contribute to excess homozygosity, leading to deviations from the Hardy–Weinberg equilibrium.^39^

The logistic regression models evaluating associations between the genetic variants—WNT11 (rs1533767) and *AXIN2* (rs3923087 and rs11867417)—and OPMD/OSCC confirmed the allelic associations, suggesting that family history of cancer and smoking habits may act as confounding variables. The A allele of WNT11 rs15337671 was associated with an increased risk for OPMD/OSCC after adjustment for family history of cancer. Similarly, the C allele of *AXIN2* SNPs (rs3923087 and rs11867417) was associated with a decreased risk for OPMD/OSCC after adjustment for smoking. In addition, family history of OC, place of residence, and alcohol consumption appeared to act as confounders in the association between *AXIN2* rs11867417 (allele C) and OPMD/OSCC.

In this study, we chose to include confirmed cases of OPMD diagnosed as oral leukoplakia and oral lichen planus for two primary reasons. First, these were the most frequently observed lesions in our clinical population during the recruitment period, and no confirmed cases of erythroplakia were recorded. While oral lichen planus is considered to have a lower malignant transformation rate,^40^ which could limit its inclusion in some studies, this may be due to variations in diagnostic criteria.^40^ In our study, participants were clinically evaluated by a single dentist with expertise in oral pathology, following the diagnostic criteria established in the WHO Classification of Head and Neck Tumours.^21^ The second reason for including these lesions is their documented involvement in dysregulation of the WNT signaling pathway, which plays a critical role in malignant transformations leading to oral cancer and is a primary focus of this study.^17^ This further justified the inclusion of oral leukoplakia and oral lichen planus.

The findings of this study should be interpreted in light of several limitations. First, the relatively small number of patients with OPMD/OSCC may have limited statistical power; this was addressed in part by carefully selecting cases and controls and using a 1:2 matching ratio. Additionally, we were unable to investigate the association among tooth agenesis, OC and OPMD/OSCC, as explained earlier. Instead, we analyzed the relationship between tooth loss or edentulism and OPMD/OSCC, with results reported separately.^31^

Genetic and environmental factors play a significant role in the etiology of both OC and oral cancer.^9^ In this study, we investigated the association between OC and OPMD/OSCC by examining environmental and genetic factors previously linked to these phenotypes. To our knowledge, this is the first study to evaluate both types of factors concurrently in exploring the etiopathogenesis of OC, OPMD, and oral cancer in a Brazilian population.

## Conclusion

No statistically significant difference was observed between cases and controls in terms of family history of orofacial clefts. However, environmental risk factors and genetic variants previously associated with orofacial cleft risk were found to be associated with oral potentially malignant disorders and oral squamous cell carcinoma. These results suggest that variations in Wnt pathway genes, along with exposure to environmental risk factors related to orofacial clefts, may modulate an individual’s susceptibility to oral potentially malignant disorders and oral cancer.

## Data Availability

Data is available on demand from the reviewers.
